# Irrespeto y maltrato durante el parto y el aborto en América Latina: revisión sistemática y metaanálisis

**DOI:** 10.26633/RPSP.2019.36

**Published:** 2019-05-03

**Authors:** Constanza Tobasía-Hege, Mariona Pinart, Sofia Madeira, Alessandra Guedes, Ludovic Reveiz, Rosario Valdez-Santiago, Vicky Pileggi, Luz Arenas-Monreal, Anabel Rojas-Carmona, Maricela Piña-Pozas, Rodolfo Gómez Ponce de León, João Paulo Souza

**Affiliations:** 1 Organización Panamericana de la Salud/Organización Mundial de la Salud (OPS/OMS) Organización Panamericana de la Salud/Organización Mundial de la Salud (OPS/OMS) WashingtonD.C. Estados Unidos de América Organización Panamericana de la Salud/Organización Mundial de la Salud (OPS/OMS), Washington D.C., Estados Unidos de América.; 2 Investigadora especialista en revisiones sistemáticas Investigadora especialista en revisiones sistemáticas Berlín Alemania Investigadora especialista en revisiones sistemáticas, Berlín, Alemania.; 3 Universidad de São Paulo (FMRP/USP) Universidad de São Paulo (FMRP/USP) Departamento de Medicina Social de la Facultad de Medicina de Ribeirão Preto São Paulo Brasil Departamento de Medicina Social de la Facultad de Medicina de Ribeirão Preto, Universidad de São Paulo (FMRP/USP), São Paulo, Brasil.; 4 Universidad de São Paulo (FMRP/USP) Universidad de São Paulo (FMRP/USP) Departamento de Pediatría de la Facultad de Medicina de Ribeirão Preto São Paulo Brasil Departamento de Pediatría de la Facultad de Medicina de Ribeirão Preto, Universidad de São Paulo (FMRP/USP), São Paulo, Brasil.; 5 Instituto Nacional de Salud Pública (INSP) Instituto Nacional de Salud Pública (INSP) Cuernavaca México Instituto Nacional de Salud Pública (INSP), Cuernavaca, México.; 6 Salud de la Mujer y Reproductiva/Organización Panamericana de la Salud (CLAP-SMR/OPS) Salud de la Mujer y Reproductiva/Organización Panamericana de la Salud (CLAP-SMR/OPS) Centro Latinoamericano de Perinatología Montevideo Uruguay Centro Latinoamericano de Perinatología, Salud de la Mujer y Reproductiva/Organización Panamericana de la Salud (CLAP-SMR/OPS), Montevideo, Uruguay.

**Keywords:** Violencia contra la mujer, parto humanizado, servicios de salud para mujeres, aborto, parto, Violence against women, humanizing delivery, women’s health services, abortion, parturition, Violência contra a mulher, parto humanizado, serviços de saúde da mulher, aborto, parto

## Abstract

**Objetivo.:**

Esta revisión sintetiza la evidencia cuantitativa, general y desglosada por categorías tipológicas de la falta de respeto y maltrato en la atención institucional del parto y el aborto en América Latina y el Caribe.

**Métodos.:**

Mediante búsquedas sistemáticas se identificaron 18 estudios primarios. Se calcularon Q e I^2^ y se realizaron metaanálisis, metarregresiones y análisis de subgrupos con la aplicación de un modelo de Der Simonian-Laird de efectos aleatorios agrupados con varianza inversa y la transformación arco-seno doble de Freeman-Tukey.

**Resultados.:**

Se identificaron estudios realizados en cinco países de América Latina. No se identificaron estudios del Caribe. La prevalencia agregada de falta de respeto y maltrato durante el parto y el aborto fue de 39%. La medida agregada para este fenómeno durante el parto fue de 43% y la medida agregada en los casos de aborto fue de 29%. La heterogeneidad elevada no permitió generar medidas agregadas según categorías tipológicas. No obstante, se presentan las frecuencias de formas específicas del fenómeno agrupadas tipológicamente.

**Conclusiones.:**

La evidencia sugiere que la falta de respeto y maltrato durante la atención del parto y el aborto son problemas de derechos humanos y salud pública prevalentes en algunos países de la Región. Es necesario lograr consenso internacional sobre la definición y operacionalización de este problema y desarrollar métodos estandarizados para su medición. Lo anterior es imprescindible para el alcance de las metas de la Agenda 2030 relacionadas con la reducción de la morbimortalidad maternoperinatal y la eliminación de todas las formas de violencia y discriminación contra la mujer.

## Abstract (en)

**Objective.:**

This review synthesizes the evidence (quantitative, general, and by typological categories) of disrespect and abuse during childbirth and abortion in health facilities in Latin America and the Caribbean.

**Methods.:**

Systematic searches identified 18 primary studies. Q and I^2^ were calculated, meta-analyses and meta-regressions were performed, and subgroups were analyzed using a DerSimonian and Laird random-effects model grouped by inverse variance and the Freeman-Tukey double arcsine transformation.

**Results.:**

Studies conducted in five Latin American countries were identified. No studies from the Caribbean were found. The aggregate prevalence of disrespect and abuse during childbirth and abortion was 39%. The aggregated prevalence of the phenomenon in childbirth was 43% and 29% during abortion. The high heterogeneity made it impossible to generate aggregate measures according to typological categories. Nevertheless, the frequencies of specific forms of the phenomenon were grouped typologically.

**Conclusions.:**

The evidence suggests that disrespect and abuse during childbirth and abortion care are human-rights and public-health problems that are prevalent in some countries of the Region. It is necessary to reach international consensus on the definition and operationalization of this problem and to develop standardized methods for its measurement. Doing so is essential in order to achieve the targets of the 2030 Agenda related to reducing maternal and newborn morbidity and mortality and eliminating all forms of violence and discrimination against women.

## RESUMO

**Objetivo.:**

Esta revisão sintetiza as evidências quantitativas, gerais e desagregadas por categorias tipológicas do desrespeito e maus-tratos na atenção institucional ao parto e ao aborto na América Latina e Caribe.

**Métodos.:**

Dezoito estudos primários foram identificados por meio de buscas sistemáticas. Foi feito o cálculo de Q e I^2^ e realizadas meta-análises, metarregressões e análises de subgrupos com um modelo de DerSimonian e Laird de efeitos aleatórios agrupados com variância inversa e transformação de Freeman-Tukey (duplo arco-seno).

**Resultados.:**

Foram identificados estudos realizados em cinco países da América Latina. Não foi identificado nenhum estudo no Caribe. Observou-se uma prevalência agregada de 39% de desrespeito e maus-tratos durante o parto e o aborto. A medida agregada para este fenômeno durante o parto foi 43% e a medida agregada nos casos de aborto foi 29%. Devido à alta heterogeneidade, não foi possível gerar medidas agregadas segundo categorias tipológicas. No entanto, são descritas as frequências de formas específicas do fenômeno agrupadas tipologicamente.

**Conclusões.:**

As evidências indicam que o desrespeito e os maus-tratos na atenção ao parto e ao aborto são uma questão de direitos humanos e de saúde pública prevalente em alguns países da Região. É preciso chegar a um consenso internacional sobre a definição e a operacionalização deste problema e elaborar métodos padronizados para mensurá-lo. Isso é imprescindível para o alcance das metas da Agenda 2030 relativas à redução da morbidade e mortalidade materna e perinatal e à eliminação de todas as formas de violência e discriminação contra a mulher.

La Organización de las Naciones Unidas ha enfatizado que la morbimortalidad materna es un problema grave de salud pública y un signo de discriminación que menoscaba el desarrollo de las naciones, y ha hecho un llamamiento a garantizar el derecho a servicios de alta calidad para la atención del embarazo y el parto y a una atención de salud digna y respetuosa para todas las mujeres (1, 2). Esto ha situado la supervivencia materno-neonatal entre los desafíos sanitarios más críticos a nivel mundial y en el núcleo de las estrategias para el logro de varias metas de los Objetivos de Desarrollo Sostenible, en especial las relacionadas con la salud materna (metas 3.1 y 3.2), y la eliminación de todas las formas de violencia y discriminación contra las mujeres (meta 5.2) (3, 4). Sin embargo, varios estudios revelan que un gran número de embarazadas en todo el mundo sufren irrespeto y maltrato en la atención institucional del parto y del aborto, períodos críticos en el curso de la vida y la salud de las mujeres (5-10).

La Organización Mundial de la Salud (OMS) emitió una declaración en la que instaba con firmeza a los sistemas de salud a erradicar dicho maltrato, que representa una violación de los derechos humanos (6, 7, 11) y del derecho de las mujeres al disfrute del nivel más alto posible de salud, perjudica la credibilidad y la confianza en los sistemas de salud, desalienta la utilización de los servicios ginecoobstétricos, disminuye la adherencia a los tratamientos y la adopción de medidas preventivas y menoscaba el bienestar integral de las mujeres y sus hijos (6, 10, 11-15), en especial en los grupos poblacionales en situación de vulnerabilidad (16).

Aunque el fenómeno ha sido reconocido desde hace varios años como un problema que afecta la atención institucional del parto y el aborto, no existe aún consenso internacional sobre su definición y operacionalización (15, 17-26) y se desconoce su magnitud a nivel mundial y regional.

A pesar de las diferentes denominaciones del fenómeno, esta revisión emplea “irrespeto y maltrato”, en consonancia con la terminología usada por la OMS (6) y contempla el parto vaginal y por cesárea y el aborto espontáneo e inducido. El objetivo es contribuir al conocimiento de su prevalencia en América Latina y el Caribe (ALC). Esto es indispensable para determinar su impacto en la salud maternoperinatal e identificar las intervenciones más efectivas para su abordaje.

## MATERIALES Y MÉTODOS

El protocolo de esta revisión sistemática está registrado y publicado en PROSPERO (CRD42016038651) (27).

Se realizó una consulta sistemática de la literatura en EMBASE, Pubmed, LILACS y Scielo de acuerdo con la estrategia de búsqueda preestablecida. Además, se revisaron en forma manual las publicaciones *Reproductive Health Matters, The Lancet, International Perspectives on Sexual and Reproductive Health* y *Revista Panamericana de Salud Pública,* por destacarse en el tema, la Región o ambos; se buscó información relevante en documentos de la Organización Panamericana de la Salud (OPS) y la OMS; se revisaron las bibliografías de los estudios incluidos y se contactaron profesionales e instituciones relevantes.

### Búsquedas

Se buscó la literatura publicada entre el 1 de enero de 1990 y el 4 de octubre de 2017. Se utilizó vocabulario controlado y con la combinación de al menos dos componentes principales: *maternal health, perinatal health, childbirth, delivery* o *abortion, obstetric* y *mistreatment of women, violence, disrespect* o *abuse*. No hubo restricción del idioma. La bibliografía se procesó utilizando EndNote, X7^®^.

### Selección de los estudios

Los títulos y resúmenes de los estudios, y los textos completos seleccionados fueron examinados por revisores independientes (MP, SM, VP, CH) mediante la aplicación de los siguientes criterios de inclusión: (i) estudios primarios o secundarios con datos cuantitativos de prevalencia de irrespeto y maltrato en la atención institucional del parto y/o aborto, (ii) revisiones sistemáticas con datos cuantitativos acerca del tema de interés, (iii) estudios producidos desde 1990 hasta las fechas de consulta de cada una de las bases de datos, (iv) estudios cuyo texto completo fuese accesible, (v) estudios publicados en inglés, español o portugués, (vi) estudios realizados en países de ALC. Se excluyeron estudios que abordaron el fenómeno fuera del ámbito institucional (por ejemplo, parto domiciliario). Las discrepancias fueron discutidas entre los revisores o examinadas por un miembro diferente del equipo hasta lograr consenso (JPS).

### Evaluación de la calidad

Se aplicó la herramienta de Hoy et al. (28) para la evaluación de riesgo de sesgo en estudios de prevalencia. Esta herramienta evalúa validez externa (criterios 1-4) e interna (criterios 5-10) bajo los siguientes dominios: sesgo de selección (criterios 1-3), sesgo de no respuesta (criterio 4), sesgo de medición (criterios 5-9) y sesgo relacionado con el análisis (criterio 10). El criterio 11 permitió calificar el riesgo como “bajo” con ocho o más criterios cumplidos, “moderado” con cinco a siete criterios y “alto” con cuatro criterios o menos. Dos miembros diferentes del equipo (MP y SM) realizaron la evaluación.

### Extracción de datos

Se utilizaron formularios estandarizados que incluían los siguientes dominios: título, autores, país, año de publicación, referencia bibliográfica, idioma original, área de cobertura, diseño, metodología, métodos de recopilación de datos, características de las participantes, tipo de parto, tipo de institución de salud, tipo de personal de salud que atendió el parto o el aborto, tipo de personal de salud asociado a los eventos, duración total de la estancia hospitalaria, tipo y frecuencia del irrespeto y maltrato (MP, SM, VP).

### Síntesis de los datos

Se extrajeron las prevalencias generales de cualquier forma de irrespeto y maltrato y se realizó combinación cuantitativa de las mismas usando la transformación arcoseno doble de Freeman-Tukey (29, 30). Se evaluaron las diferencias de prevalencia según el tipo de hospital, método de recolección de datos y tipo de atención obstétrica mediante el cálculo de la proporción del fenómeno para cada estudio junto con los intervalos de confianza asociados del 95% (IC95%) y con la utilización de un modelo de DerSimonian-Laird de efectos aleatorios agrupados con el método de varianza inversa. Los análisis estadísticos se realizaron con R.

Además, se extrajeron las prevalencias específicas del fenómeno usando como referencia la clasificación tipológica de maltrato durante el parto descrita por Bohren et al. (8) que consta de siete categorías tipológicas (términos de tercer orden), desglosadas a su vez en subcategorías analíticas (términos de segundo orden) y descriptivas (términos de primer orden). Estas prevalencias desglosadas por tipo no fueron metaanalizadas.

### Evaluación de la heterogeneidad

La proporción de varianza debida a la heterogeneidad estadística se calculó usando Q e I^2^. I^2^ menor de 25% representó heterogeneidad baja; entre 25% y 50%, moderada; entre 51% y 75%, sustancial, y entre 76% y 100%, elevada. La evaluación de las posibles fuentes de heterogeneidad se realizó mediante análisis de subgrupo y metarregresiones de efectos aleatorios univariadas por país: Brasil versus otros países, tipo de parto, tipo de hospital, método de recolección de datos y tipo de desenlace medido (trato deshumanizante e insatisfacción con la atención recibida vs. ocurrencia de alguna forma de irrespeto y maltrato). No se encontró una variable estadísticamente significativa para realizar metarregresiones multivariadas.

El informe de resultados siguió las pautas del PRISMA (*Preferred Reporting Items for Systematic Review and Meta-Analysis*) (31).

## RESULTADOS

**FIGURA 1:**
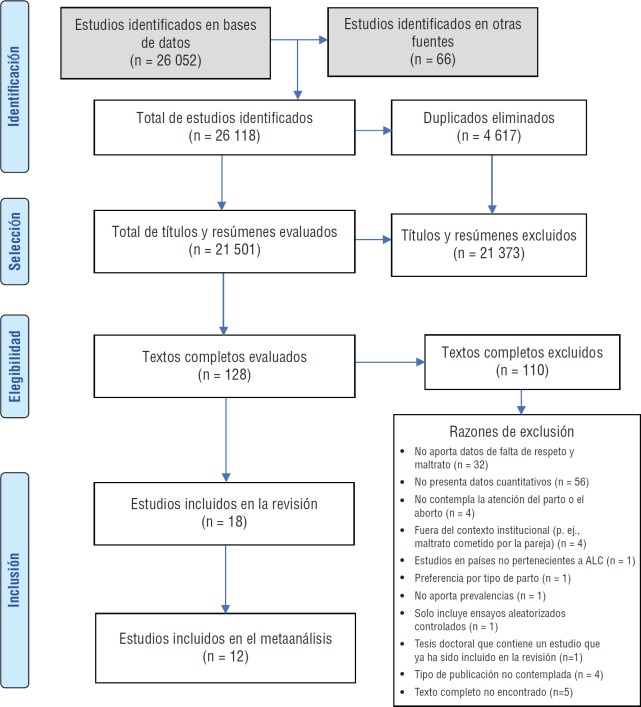
Diagrama de flujo del proceso de búsqueda e inclusión de estudios

Las búsquedas en las bases de datos y en otras fuentes arrojaron 26 118 estudios. Tras eliminar duplicados y analizar títulos y resúmenes, se seleccionaron 128 estudios para lectura completa. Se incluyeron un total de 18 estudios primarios (15, 20, 21, 23, 25, 32-44), de los cuales 12 se incluyeron en el metaanálisis (figura 1).

Los estudios se realizaron en cinco países: 12 estudios en Brasil (15, 20, 23, 25, 32, 33, 36, 38-42), un estudio en Chile (37), dos estudios en México (34, 35), un estudio en Perú (43) y dos estudios en Venezuela (21, 44). No se encontraron estudios desarrollados en el Caribe. Los trabajos fueron publicados entre 2005 y 2017 y, con excepción de uno de casos y controles (42), todos los estudios fueron de corte transversal. El tamaño de la muestra varió entre 78 (20) y 15 688 mujeres (36), con un total de 37 028. Catorce investigaciones exploraron el fenómeno de interés en el contexto del parto vaginal y por cesárea. Dos estudios exploraron solo el aborto inducido (20, 41) y dos más abarcaron el parto vaginal y por cesárea y el aborto inducido y espontáneo (33, 44). Los métodos para la recolección de datos fueron entrevistas y revisiones del historial clínico de las mujeres (cuadro 1).

El riesgo de sesgo fue clasificado como bajo para tres estudios (17%), moderado para nueve (50%) y alto para seis (33%). Ningún estudio satisfizo los diez criterios evaluados.

ALC, América Latina y el Caribe.

**CUADRO 1:** Características de los estudios incluidos

Estudio	País	Diseño del estudio	Duración de la recolección de datos	Método de muestreo	Tamaño de la muestra (N)	Instrumentos de medida	Tipo de parto o aborto	IM/I (n)	Prevalencia (%)
**Andrade 2016 (23)**	Brasil	Transversal	Agosto a diciembre de 2014	Todas las mujeres que tuvieron parto vaginal durante el de estudio en un hospital de referencia de Recife	603	Entrevistas e historiales médicos de la mujer y del recién nacido	Vaginal (n = 591), vaginal instrumental (n = 12)	522	86,6
**Aquino 2012 (41)**	Brasil	Transversal	Agosto a diciembre de 2010	Censo de mujeres internadas en siete hospitales de Salvador, ocho de Recife y cuatro de San Luis. Se incluyeron todos los hospitales públicos con internaciones por aborto	2 804	Entrevistas cara a cara usando cuestionarios validados	Aborto inducido	235	8,4
**Binfa 2016 (37)**	Chile	Transversal	Setiembre de 2010 a junio de 2011	Se seleccionaron mujeres (método no descrito) en dos hospitales principales pertenecientes al sistema nacional de salud del área metropolitana de Santiago	508	Entrevistas cara a cara mediante el uso de cuestionarios validados	Parto vaginal (n = 401) y cesárea (n = 107)	141^a^	27,8
**Busanello 2011 (38)**	Brasil	Transversal	Julio de 2008 a febrero de 2009	Todo el personal sanitario del centro obstétrico de un hospital universitario e historiales médicos de todas las adolescentes que parieron durante el periodo de estudio	128 adolescentes y 23 trabajadores	Entrevistas semiestructuradas (trabajadores) y revisión de historiales médicos (adolescentes)	Parto vaginal	NRD	NRD
**D’Orsi 2005 (42)**	Brasil	Caso-control	Octubre de 1998 a marzo de 1999	Conveniencia. Se seleccionaron el 50% de partos vaginales y todos los partos por cesárea ocurridos el día anterior a la entrevista Dos maternidades, una pública y otra privada dentro del sistema nacional de salud	909	Entrevistas e historiales médicos de la mujer	Parto vaginal (n = 454) y cesárea (n = 455)	NRD	NRD
**D’Orsi 2014 (36)**	Brasil	Transversal	Marzo de 2011 a febrero de 2013	Se seleccionaron hospitales, tanto públicos como privados, con más de 500 partos/año	15 688	Entrevistas telefónicas y cara a cara con cuestionarios validados e historiales médicos de las mujeres	Parto vaginal (44,6%) y cesárea (53,4%)	922	5,9
**Madeiro 2017 (20)**	Brasil	Transversal	Junio de 2012 a noviembre de 2013	Todas las mujeres internadas por aborto incompleto en el hospital público de referencia de Teresina, Piauí	78	Entrevista semiestructurada grabada siguiendo un cuestionario validado	Aborto inducido	26	33,3
**Montesinos Segura 2017 (43)**	Perú	Transversal	Abril a julio 2016	El objetivo original fue el de reclutar todos los hospitales de todas las ciudades con un gran número de habitantes Sólo se obtuvieron permisos de 14 hospitales públicos localizados en nueve ciudades del Perú	1 528	Cuestionario de 36 ítems (Bowser y Hill y otros estudios), aplicado a a mujeres en el posparto inmediato (se realizó una prueba piloto para evaluar los instrumentos)	Parto vaginal (n = 968) y cesárea (n = 560)	1 488	97,4
**Nagahama 2008 (39)**	Brasil	Transversal	Marzo de 2005 a febrero de 2006	Censo de partos vivos en dos hospitales del Sistema Único de Salud (SUS) durante el período de estudio	569	Entrevistas con preguntas abiertas y cerradas (de cuestionarios validados)	Parto vaginal (n = 273) y cesárea (n = 296)	NRD	NRD
**Narchi 2010 (32)**	Brasil	Transversal	Enero a julio de 2008	Conveniencia. Entre usuarias atendidas en dos instituciones públicas de salud de la zona oriental de la ciudad de San Pablo	90	Entrevistas con preguntas abiertas y cerradas (basadas en instrumentos validados) además de revisión de historiales médicos	Parto vaginal (n = 82) y cesárea (n = 8)	76^a^	84,4
**Nieto-González 2011 (34)**	México	Transversal	En 2010 (durante ocho semanas)	Muestra representativa (método no descrito) de mujeres embarazadas de segunda gesta de más de 22 semanas de embarazo obtenidas del promedio mensual de mujeres que acuden a la consulta prenatal de un hospital general público de Ciudad de México	380	Encuestas autoaplicadas a mujeres que voluntariamente accedieron a participar en el estudio basadas en instrumentos validados	Tipo de parto reportado	23	6,1
**Pereira 2015 (44)**	Venezuela	Transversal	Junio a octubre de 2012	Muestreo de carácter intencional no probabilístico. Se incluyeron mujeres hospitalizadas en el departamento de obstetricia y ginecología del Hospital General del Oeste “Dr. José Gregorio Hernández” (público) de Caracas, Venezuela, para ser atendidas por parto, cesárea o por presentar aborto espontáneo	326	Entrevista con un cuestionario (no validado) de 15 preguntas cerradas para saber si fueron sometidas a cualquier tipo de agresión física o psicológica	Partos (n = 225, vaginal y cesárea) y aborto espontáneo (n = 101)	86^b^	26,4^c^
**Rodrigues 2017 (25)**	Brasil	Transversal	Noviembre 2013 a enero 2014	La Red Cigüeña fue implantada por el Ministerio de Salud en 2011 en hospitales públicos pertenecientes al sistema único de salud. En Fortaleza-Cascavel, la Red Cigueña está compuesta por 11 hospitales-maternidades de media y alta complejidad	293^d^	Cuestionario aplicado a mujeres en el posparto además de revisión de historiales médicos	Parto vaginal	NRD	NRD
**Santos 2008 (40)**	Brasil	Transversal	Julio a diciembre de 2006	Todas las mujeres que parieron en un hospital público del interior de San Pablo durante el período de estudio	279	Historiales médicos	Parto vaginal	NRD	NRD
**Souza 2017 (15)**	Brasil	Transversal	Durante 2011	Muestreo por conglomerados en etapas múltiples Se seleccionaron 25 centros de salud en 21 de las 30 regiones administrativas del distrito federal	10 468	Entrevista con cuestionario validado	Parto vaginal (n = 2 774) y cesárea (n = 7 558)	^a^	2,6
**Terán 2013 (21)**	Venezuela	Transversal	Mayo a agosto de 2011	Selección (no reporta el método de selección) de usuarias ingresadas en los servicios de puerperio de la Maternidad “Concepción Palacios”	425	Encuestas autoaplicadas contestadas de forma anónima	Puerperio de parto simple (n = 249), parto instrumental (n = 9), cesárea (n = 167)	210	49,4
**Valdez-Santiago 2013 (35)**	México	Transversal	Mayo a junio de 2012	Método no reportado Se seleccionaron usuarias de tres hospitales públicos del estado de Morelos: Hospital General de Tetecala, Hospital de la Mujer de Yautepec y Hospital General José G. Parres de Cuernavaca	512	Cuestionario aplicado a mujeres en el posparto inmediato (se realizó una prueba piloto para evaluar los instrumentos)	Parto vaginal (42,6%), cesárea (50%) y cesárea programada (7,4%)	149	29,1
**Venturi 2013 (33)**	Brasil	Transversal	Durante agosto 2010	Mujeres atendidas en hospitales públicos y privados de 25 estados de Brasil durante el período de estudio	1 411	Encuestas basadas en instrumentos validados	Parto (tipo no reportado)	353	25,0
					28		Aborto inducido	15	53,6

IM/I (n), falta de respeto y maltrato/insatisfacción (número de eventos); NRD, no se reportaron datos.

a Descontento o insatisfacción con el trato recibido.

b De los cuales: parto (n = 36), cesárea (n = 19) y aborto (n = 31).

c Partos (55/225 = 24,4%) y abortos (31/101 = 30,7%)

d Rodrigues reclutó 3 765 mujeres, pero solo reportó datos de 293.

**CUADRO 2:** Prevalencias generales y específicas de irrespeto y maltrato en la atención institucional del parto^a^

		Andrade 2016 [23]	Binfa 2016 [37]	Busanello 2011 [38]	D’Orsi 2005 [42]	D’Orsi 2014 [36]	Montesinos Segura 2017 [43]	Nagahama 2008 [39]	Narchi 2010 [32]	Nieto-González 2011 [34]	Pereira 2015 [44]	Rodrigues 2017 [25]	Santos 2008 [40]	Souza 2017 [15]	Terán 2013 [21]	Valdez-Santiago 2013 [35]	Venturi 2013 [33]
		n (%)	n (%)	n (%)	n (%)	n (%)	n (%)	n (%)	n (%)	n (%)	n (%)	n (%)	n (%)	n (%)	n (%)	n (%)	n (%)
**Tamaño muestra (N)**		603	508	128	909	15 688	1 528	569	90	380	225	293	279	10 468	425	512	1 411
Algún tipo de falta de respeto y/o insatisfacción/descontento (%)		522 (86,6)	141 (27,8)	NRD	NRD	922 (5,9)	1488 (97,4)	NRD	76 (84,4)	23 (6,1)	55 (26,4)	NRD	NRD	NRD (2,6)	210 (49,4)	149 (29,1)	353 (25,0)
**Tipo de maltrato**																	
**Abuso físico**																	
Uso de la fuerza																	
	Empujones	NRD	NRD	NRD	NRD	233^b^ (1,5)	NRD	NRD	NRD	NRD	NRD	NRD	NRD	NRD	NRD	NRD	4 (0,3)
	Golpes en las piernas, pellizcos, presión en el abdomen, amenazas	NRD	NRD	NRD	NRD	NRD	24 (1,6)	NRD	NRD	NRD	NRD	NRD	NRD	NRD	NRD	39 (7,6)	4 (0,3)
Restricción física																	
	Restricción física a la cama o amordazamiento durante el parto	NRD	NRD	NRD	NRD	NRD	52 (3,4)	NRD	NRD	NRD	NRD	NRD	NRD	NRD	NRD	NRD	4 (0,3)
**Abuso sexual**																	
Abuso sexual																	
	Acoso sexual	NRD	NRD	NRD	NRD	NRD	11 (0,7)	NRD	NRD	NRD	NRD	NRD	NRD	NRD	NRD	NRD	4 (0,3)
**Abuso verbal**						360 (2,3)										99^c^ (19,3)	
Lenguaje rudo																	
	Lenguaje rudo/gritos	NRD	NRD	NRD	NRD	NRD	NRD	NRD	NRD	NRD	NRD	**11 (3,8)**	NRD	NRD	NRD	NRD	32 (2,3)
Amenazas y culpa																	
	Amenazas	NRD	NRD	NRD	NRD	393^d^ (2,5)	NRD	NRD	NRD	NRD	NRD	24 (8,2)	NRD	NRD	NRD	NRD	NRD
	Culpar a las mujeres de los malos resultados	NRD	NRD	NRD	NRD	Mencionado	NRD	NRD	NRD	NRD	NRD	NRD	NRD	NRD	NRD	Mencionado	NRD
	Amenazas de suspensión del tratamiento o de malos resultados	NRD	NRD	NRD	NRD	Mencionado	NRD	NRD	NRD	NRD	NRD	NRD	NRD	NRD	NRD	Mencionado	NRD
**Discriminación**																	
Discriminación basada en condiciones médicas																	
	Discriminación basada en la presencia de infecciones de transmisión sexual	NRD	NRD	NRD	NRD	NRD	22 (1,4)	NRD	NRD	NRD	NRD	NRD	NRD	NRD	NRD	NRD	NRD
Discriminación por razones socioeconómicas																	
	Discriminación por razones socioeconómicas	NRD	NRD	NRD	NRD	NRD	482 (31,5)	NRD	NRD	NRD	NRD	NRD	NRD	NRD	NRD	NRD	NRD
	Discriminación por razones de edad	NRD	NRD	NRD	NRD	NRD	70 (4,6)	NRD	NRD	NRD	NRD	NRD	NRD	NRD	NRD	NRD	NRD
	Discriminación basada en estado civil	NRD	NRD	NRD	NRD	NRD	49 (3,2)	NRD	NRD	NRD	NRD	NRD	NRD	NRD	NRD	NRD	NRD
	Discriminación basada en la religión	NRD	NRD	NRD	NRD	NRD	32 (2,1)	NRD	NRD	NRD	NRD	NRD	NRD	NRD	NRD	NRD	NRD
**Incumplimiento de las normas profesionales de atención**																	
Falta de consentimiento informado y confidencialidad																	
	Falta de consentimiento informado y confidencialidad	NRD	NRD	NRD	NRD	NRD	1 037 (68,1)	NRD	NRD	NRD	NRD	NRD	NRD	NRD	NRD	NRD	NRD
Exámenes y procedimientos físicos																	
	Exámenes y procedimientos físicos	NRD	NRD	NRD	NRD	NRD	1029 (67,6)	NRD	NRD	NRD	NRD	NRD	NRD	NRD	NRD	NRD	NRD
	Tactos vaginales dolorosos	NRD	NRD	NRD	NRD	233 (27,7) (además de empujones)	NRD	NRD	NRD	NRD	NRD	NRD	NRD	NRD (6,9)	NRD	NRD	35 (2,5)
	Negativa a proporcionar alivio del dolor/ausencia de analgesia	NRD	NRD	NRD	NRD	393 (2,5)^e^	142 (9,3)	NRD	55 (61,1)	NRD	NRD	NRD	NRD	NRD (6)	NRD	NRD	35 (2,5)
	Sutura sin anestesia/revisión uterina sin anestesia	NRD	NRD	NRD	NRD	NRD	78 (5,1)	NRD	NRD	NRD	NRD	49 (16,8)	NRD	NRD	60 (14,1)	39 (7,6) entre otros abusos físicos	NRD
	Procedimientos quirúrgicos sin consentimiento/sin información	NRD	NRD	NRD	NRD	NRD	1101 (74,6)	NRD	NRD	NRD	NRD	109 (37,2)	NRD	NRD (3,6)	284 (66,8)	NRD	32 (2,3)
	Infusión venosa de rutina durante el trabajo de parto	NRD	507 (99,8)	NRD	527 (58,8)	NRD	15 (1,0)	NRD	NRD	NRD	NRD	187 (63,8)	NRD	NRD	NRD	NRD	NRD
	Uso de la posición supina/decúbito dorsal	162 (26,9)	NRD	NRD	718 (87,0)	NRD	NRD	NRD	NRD	NRD	NRD	NRD	NRD	NRD	106 (24,9)	NRD	NRD
	Uso de la posición litotómica para el parto	72 (11,9)	400 (86,0)	NRD	446 (98,5)	NRD	NRD	NRD	NRD	NRD	NRD	NRD	NRD	NRD	NRD	NRD	NRD
	Examen rectal	59 (9,8)	NRD	NRD		NRD	NRD	NRD	NRD	NRD	NRD	NRD	NRD	NRD	NRD	NRD	NRD
	Administración de oxitócicos	245 (40,6)	NRD	NRD (13,3)	335 (37,4)	NRD	219 (14,3)	NRD	NRD	NRD	NRD	101 (34,5)	NRD	NRD	133 (31,3)	NRD (55)	NRD
	Presión para pujar	390 (64,7)	NRD	NRD	NRD	NRD	NRD	NRD	NRD	NRD	NRD	NRD	NRD	NRD	NRD	NRD	NRD
	Amniotomía precoz	186 (30,9)	NRD	NRD	155 (17,7)	NRD	NRD	NRD	NRD	NRD	NRD	97 (33,3)	NRD	NRD	NRD	NRD	NRD
	Maniobra de Kristeller	52 (8,6)	NRD	NRD	225 (49,8)	NRD	306 (20,0)	NRD	NRD	NRD	NRD	70 (24,1)	NRD	NRD	67 (15,8)	NRD	NRD
	Maniobra de Valsalva	NRD	NRD	NRD	NRD	NRD	NRD	NRD	NRD	NRD	NRD	254 (86,7)	NRD	NRD	NRD	NRD	NRD
	Manipulación activa del feto / tactos vaginales repetidos o no consentidos	112 (18,6)	NRD	NRD	280 (36,9)	NRD	864 (57,7)	NRD	NRD	NRD	NRD	152 (52,2)	NRD	NRD	158 (37,2)	NRD (98)^f^	NRD
	Uso de la episiotomía	13 (2,2)	255 (57,5)	NRD (46,1)	368 (81,4)	NRD	100 (6,6)	NRD	NRD	NRD	NRD	60 (20,5)	214 (87,0)		85 (20)	NRD (66,5)	NRD
	Pinzamiento precoz del cordón umbilical	180 (29,9)	NRD	NRD	NRD	NRD	NRD	NRD	NRD	NRD	NRD	NRD	NRD	NRD	NRD	NRD	NRD
Descuido y abandono																	
	Negligencia, abandono o retrasos en la atención	NRD	NRD	NRD	NRD	NRD	347 (22,7)	NRD	NRD	NRD	NRD	NRD	NRD	NRD (2,4)^g^	NRD	NRD	28 (2,0)
	Ausencia de proveedor entrenado para la atención del parto	NRD	NRD	NRD	NRD	NRD	NRD	NRD	NRD	221 (58,2)	NRD	185 (63,1)	NRD	NRD	NRD	NRD	NRD
**Problemas de relación entre mujeres y proveedores de salud**																	
Comunicación inefectiva																	
	Mala comunicación/imposibilidad de manifestar miedos, inquietudes e inseguridades	NRD	NRD	NRD	NRD	NRD	NRD	NRD	3 (3,3)	79 (20,8)	NRD	NRD	NRD	NRD	83 (19,5)	NRD	NRD
	No tomar en cuenta las preocupaciones de las mujeres	NRD	NRD	NRD	NRD	NRD	NRD	NRD	NRD	31 (8,2)	NRD	NRD	NRD	NRD	NRD	NRD	
	Humillaciones/mala actitud del personal hacia las mujeres	NRD	NRD	NRD	NRD	393 (2,5)^e^	145 (9,5)	NRD	14 (15,6)	23 (6,1) no fueron respetadas Enfermeras que: no se dirigían por su nombre 234 (61,6); no eran amables 37 (9,7)	NRD	Bromas: 10 (3,4); Uso de motes: 72 (24,6)	NRD	NRD	Pacientes escucharon comentarios irónicos o chistes 65 (15,3); diminutivos 44 (10,4); recibieron críticas por llorar o gritar 92 NRD (21,6)	NRD (19)	25 (1,8)
Falta de apoyo y atención																	
	Falta de atención y apoyo de los proveedores de salud	NRD	NRD	NRD	NRD	393 (2,5)^e^	NRD	NRD	NRD	34 (8,9)	NRD	NRD	NRD	NRD	NRD	NRD (19)	NRD
	Negación o ausencia de acompañante	NRD	169 (33,3)	NRD	812 (90,5)	NRD	429 (28,1)	NRD (26,3)	1 (1,1)	NRD	NRD	Sin acompañante: 114 (39,3); sin acompañante durante el tiempo de interación:41 (22,4)	NRD	NRD (25,6)	NRD	NRD	NRD
	Negación o restricción de alimentos, líquidos o movilidad	NRD	393 (77,4)	NRD	NRD	NRD	Alimentos: 213 (13,9) Movilidad: 335 (21,9)	NRD	NRD	NRD	NRD	Alimentos: 225 (77,3); líquidos: 208 (70,8): movilidad:110 (37,5)	NRD	NRD	67 (15,8)	NRD	NRD
	Falta de respeto a las posiciones de parto preferidas por las mujeres	NRD	NRD	NRD	NRD	NRD	93 (6,1)	NRD	NRD	NRD	NRD	NRD	NRD	NRD	49 (11,5)	NRD	NRD
	Detención en el establecimiento	NRD	NRD	NRD	NRD	NRD	3 (0,2)	NRD	NRD	NRD	NRD	NRD	NRD	NRD	NRD	NRD	NRD
**Condiciones y limitaciones de los establecimientos de salud**																	
Falta de recursos																	
	Carencia de privacidad	NRD	NRD (100)	NRD	NRD	NRD	Ausencia de cortinas: 922 (60,4) Demasiado personal en la sala de parto: 70 (4,6)	NRD	3 (3,3)	220 (57,9)	NRD	NRD	NRD	NRD	NRD	NRD	NRD

NRD: no se reportaron datos.

a Las prevalencias específicas se muestran clasificadas según categorías tipológicas de Bohren et al. (8). Se muestran solo las categorías y subcategorías para las cuales se reportaron datos.

b Incluye también daños o tactos vaginales dolorosos.

c Incluye frases como “no grite”, “no llore”, “no se queje”, “regaños”, “humillaciones”, además en algunos casos reportaron ser “ignoradas” por el personal que las atendió.

d Incluye también humillaciones, negación de atención médica y negación de proporción de alivio para el dolor, y lo engloban como abuso psicológico.

e Analizaron la falta de respeto y el maltrato psicológicos, que contenía humillaciones, falta de alivio para el dolor y negación de atención médica; Pereira 2015 (44): una misma pregunta engloba amenazas, gritos y regaño.

f Tuvieron un promedio de 5 ± 3 tactos vaginales (rango 1-20).

g La negligencia fue definida como la negación de alivio para el dolor, procedimientos quirúrgicos sin explicaciones y la negación de asistencia.

**CUADRO 3:** Prevalencias generales y específicas de irrespeto y maltrato en la atención institucional del aborto^a^

Tipo de malos tratos		Aquino 2012 (41) (n = 2 804)		Madeiro 2017 (20) (n = 78)		Pereira 2015^b^ (44) (n = 101)		Venturi 2013^c^ (33) (n = 28 reportaron aborto)	
		n	%	n	%	n	%	n	%
Algún tipo de falta de respeto o insatisfacción		235^d^	8,4	26	33,6	31	30,7	15	53,6
**Abuso verbal**									
Lenguaje rudo									
	Lenguaje rudo y grosero	NRD	NRD	18	23,1	NRD	NRD	NRD	NRD
	Reprimenda/gritos	NRD	NRD	12	15,4	NRD	NRD	NRD	NRD
	Comentarios acusatorios o basados en prejuicios	NRD	NRD	NRD	NRD	NRD	NRD	1	3,6
Amenazas y culpa									
	Amenaza de denuncia a la policía	NRD	NRD	22	28,2	NRD	NRD	5	17,9
**Estigma y discriminación**									
Discriminación basada en condiciones médicas									
	Juicio moral por la práctica de aborto o tratadas como sospechosas o criminales	157	5,6	24	30,8	NRD	NRD	10	35,7
**Incumplimiento de las normas profesionales de atención**									
Falta de consentimiento informado y confidencialidad									
	Divulgación del historial médico sin consentimiento	NRD	NRD	12	15,4	NRD	NRD	NRD	NRD
Exámenes y procedimientos físicos									
	Negativa a proporcionar alivio del dolor o ausencia de analgesia	49	1,8	20	25,6	NRD	NRD	NRD	NRD
	Procedimientos quirúrgicos sin consentimiento	NRD	NRD	15	19,2	NRD	NRD	6	21,4
	Tacto vaginal sin consentimiento	NRD	NRD	10	12,8	NRD	NRD	NRD	NRD
	Transfusión sanguínea sin consentimiento	NRD	NRD	4	5,1	NRD	NRD	NRD	NRD
	Histerectomía sin consentimiento	NRD	NRD	1	1,3	NRD	NRD	NRD	NRD
Descuido y abandono									
	Retraso en ser atendidas	NRD	NRD	22	28,2	NRD	NRD	5	17,9
**Problemas de relación entre mujeres y proveedores de salud**									
Falta de apoyo y atención									
	Ausencia de acompañante	Salvador Recife San Luis	49,2 13,8 71,2	20	25,6	NRD	NRD	NRD	NRD
**Condiciones y limitaciones del sistema de salud**									
Falta de recursos									
	Internación conjunta con otras puérperas sin privacidad	NRD	NRD	21	26,9	NRD	NRD	NRD	NRD
	Entrevista y examen físico realizado en presencia de otras parteras	NRD	NRD	12	15,4	NRD	NRD	NRD	NRD
	Falta de suministros médicos (torundas absorbentes)	Salvador Recife San Luis	11,8 37,0 80,4	NRD	NRD	NRD	NRD	NRD	NRD
Insuficiente cambio de sábanas		Salvador Recife San Luis	30,3 46,1 60,2	NRD	NRD	NRD	NRD	NRD	NRD

a Las prevalencias específicas se muestran según categorías tipológicas de Bohren et al. (8). Se muestran solo las categorías y subcategorías para las cuales se reportaron datos.

b Los datos según las distintas categorías fueron reportados de manera agregada para partos y abortos (cuadro 2).

c En ese estudio 95 mujeres reportaron situación de aborto provocado, pero solo 28 buscaron atención médica. El denominador escogido es por tanto el de mujeres que acudieron al hospital.

d 235 mujeres se sintieron discriminadas.

Las características sociodemográficas de las mujeres encuestadas se describieron en forma limitada. Solo dos estudios (36, 44) documentaron la prevalencia general de algún tipo de irrespeto y maltrato según características sociodemográficas. Diez estudios (56%) reportaron la procedencia de las mujeres (entorno rural o urbano), nueve estudios (50%) reportaron estado civil, 15 (83%) aportaron información sobre el nivel educativo de las encuestadas, seis (33%) documentaron la ocupación, siete (39%) la etnia y nueve (50%) proporcionaron datos sobre la paridad. Catorce estudios (78%) se desarrollaron en hospitales urbanos públicos, de los cuales dos fueron universitarios. Cuatro estudios (22%) incluyeron mujeres tanto de hospitales públicos como privados, aunque los desenlaces de interés no se reportaron por separado. Solo dos estudios (11%) incluyeron información específica sobre el personal que brindó la atención, que señala que la mayoría fueron profesionales de medicina.

Las definiciones operativas variaron de manera sustancial entre los estudios. Los equipos de investigación usaron criterios derivados de las prácticas para el parto normal de la OMS (45), del panorama analítico propuesto por Bowser y Hill (46) y de escalas diseñadas para evaluar satisfacción con la atención.

Sin embargo, doce estudios reportaron prevalencias generales de irrespeto y maltrato medidas como la ocurrencia de alguna forma de abuso verbal, físico o ambos, trato deshumanizante o insatisfacción con la atención (20, 21, 23, 32-37, 41, 43, 44) las cuales fueron metaanalizadas. Además, la mayoría de los estudios (15, 20, 21, 23, 25, 32-43) reportó prevalencias específicas, las cuales se agruparon por categorías tipológicas. No se realizó metaanálisis de prevalencias específicas (cuadros 2 y 3).

La medida agregada de la prevalencia general de irrespeto y maltrato en el parto y el aborto conjuntamente resultó en 39% (IC95%: 19-62%, I^2^ = 99,9%). Esta medida no difirió de manera significativa según el tipo de atención obstétrica (parto versus aborto, Q = 0,54, *P* = 0,46), el tipo de hospital (público versus ambos, público y privado, Q = 1,14, *P* = 0,29), el método de recopilación de datos (solo entrevista versus entrevistas y revisión del historial clínico, Q = 0,33, *P* = 0,56), ni la calidad de los estudios (riesgo de sesgo bajo versus moderado versus alto, Q = 1,12, *P* = 0,57).

La medida agregada de la prevalencia durante la atención institucional del parto (vaginal y cesárea) fue de 43% (IC95%: 15-74%, I^2^ = 99,9%). Esta medida no difirió de manera significativa según el tipo de hospital (Q = 3,76, *P* = 0,05), el método de recopilación de datos (Q = 0,24, *P* = 0,62), ni la calidad de los estudios (Q = 2,34, *P* = 0,31). La misma medida en los casos de aborto (espontáneo e inducido, n = 4) fue de 29% (IC95%: 10-53%, I^2^ = 96,8%). Esta estimación no difirió significativamente según el tipo de aborto (espontáneo vs inducido, Q = 0,02, *P* = 0,90).

La heterogeneidad resultó elevada (I^2^ > 90%, *P* < 0,001). Ninguna de las variables usadas en las metarregresiones explicó este valor. Por lo tanto, se debe tener precaución a la hora de interpretar los hallazgos de los metaanálisis, ya que la alta variabilidad puede estar asociada a la disminución de la calidad de la evidencia y afectar la confiabilidad de los resultados.

Con respecto a las prevalencias específicas, se documentaron frecuencias para todas las categorías analíticas y la mayoría de las subcategorías descriptivas, si bien ninguno de los estudios por sí solo reportó datos para todas ellas. El incumplimiento de los estándares profesionales de la atención y la dificultad en la relación entre los proveedores de atención y las mujeres fueron las dos categorías más documentadas. Con relación a la primera categoría, se documentaron prevalencias entre 2,5% y 27% (33, 36) para tactos vaginales dolorosos y entre 19% (20) y 74,6% (43) para la falta de consentimiento informado.

En los casos de parto, la falta de consentimiento para la ligadura tubárica se documentó en 7% y, para la histerectomía, en 4% (43). En los casos de aborto, la falta de consentimiento para la transfusión sanguínea y la histerectomía se reportaron en 5,1% y 15,3%, respectivamente (20), y para la episiotomía, entre 2% (23) y 87% (40). El uso sistemático de oxitócicos osciló entre 13% (38) y 41% (23), la amniotomía precoz habitual entre 17,7% (23) y 33,3% (25) y la negativa en el uso de medicamentos para aliviar el dolor fluctuó entre 2% (41) y 61% (32). Por su parte, la prevalencia de la maniobra de Kristeller varió entre 8,6% (23) y 50% (43) y la de Valsalva se documentó en 87% (25). La falta de privacidad en el parto y el aborto fue documentada entre 27% (20) y 100% (37).

En el ámbito de la relación de las mujeres con los proveedores, la negación de un acompañante durante el parto o el aborto fue reportada por ocho estudios con frecuencias que variaron ampliamente entre 1% (32) y 91% (42). Siete estudios documentaron trato humillante entre 2% (33) y 62% (34); y tres, restricciones de la movilidad entre 8% (21) y 75% (37). Otras formas de abuso en esta categoría incluyeron burlas, críticas por llorar, no ser llamadas por el nombre, uso de apodos, no tomar en cuenta las preocupaciones de la mujer, negación de alimentos o líquidos y restricción de la movilidad. Solo un estudio documentó discriminación durante el parto (43) con frecuencia de 35% en hospitales públicos rurales, principalmente. En contraste, esta forma de abuso se reportó en todos los estudios con datos sobre aborto (20, 33, 41) con prevalencias entre 5,6% y 35,7% (20, 33). Dos de ellos con frecuencia superior a 30% (20, 31).

## DISCUSIÓN

Los 18 estudios incluidos en esta revisión y las medidas documentadas sugieren que el irrespeto y el maltrato son problemas prevalentes en la atención institucional del parto y el aborto en América Latina. Más de un tercio de las mujeres entrevistadas informaron haber sufrido alguna forma de irrespeto o maltrato o tener descontento con la atención recibida; y miles reportaron alguna forma de abuso físico o verbal, discriminación, incumplimiento de las normas profesionales de la atención, problemas en la relación con los proveedores de atención y derivados de las limitaciones logísticas o del funcionamiento de los establecimientos de salud.

La complejidad del fenómeno, la dificultad para establecer una definición consensuada sobre qué lo constituye, así como la falta de métodos de medición validados y comparables (47) pueden estar asociados con la escasez de los estudios sobre este tópico en la Región, la marcada variabilidad y la dificultad para la síntesis de los datos. El enfoque de algunos estudios en formas de irrespeto y maltrato con mayor prevalencia y de otros en aquellas menos prevalentes, así como la cantidad de formas del fenómeno que se incluyen en los estudios, pueden contribuir a la elevada heterogeneidad.

Además, mientras algunas formas identificadas como irrespeto y maltrato son bien reconocidas como tal (p. ej., amenazas o falta de consentimiento para procedimientos médicos), aún hay controversia sobre el reconocimiento de otras formas (p. ej., la ausencia de privacidad o la aplicación de intervenciones no basadas en evidencia científica), lo cual tiene implicaciones para la producción científica. Esto ratifica la necesidad de tener precaución a la hora de interpretar, comparar y concluir sobre los hallazgos presentados, así como la de aumentar el acervo de evidencia con metodologías estandarizadas y datos comparables que informen el desarrollo de intervenciones efectivas para el abordaje de esta problemática.

Los estudios cuantitativos sobre este tema en países de altos ingresos son escasos. No obstante, se han documentado altas variabilidades en la prevalencia también en trabajos desarrollados en África y Asia, cuyas medidas fluctúan entre 15% y 98% (48-50). Los estudios africanos muestran frecuencias más altas de discriminación, procedimientos sin consentimiento y, además, retención de las mujeres en instituciones hospitalarias por falta de pago.

Las exploraciones relevantes sobre los factores de riesgo y las causas de este fenómeno quedan fuera del alcance de esta revisión. Sin embargo, los modelos de formación profesional jerárquicos y débiles en competencias para la comunicación y las habilidades médico-paciente, el desempoderamiento de las mujeres, la falta de personal y de programas de educación continuada, las deficiencias logísticas, así como la ausencia de protocolos estandarizados de atención y la carencia de mecanismos de control de calidad y de rendición de cuentas en las instituciones de salud pueden estar dentro de las posibles causas del fenómeno.

Aunque los estudios de irrespeto y maltrato se han enfocado sobre todo en el parto, es importante incentivar la inclusión del aborto en estas investigaciones, ya que tres de los cuatro estudios sobre aborto describieron prácticas discriminatorias como el juicio moral, el trato de las mujeres como sospechosas o criminales, amenazas de denuncia a la policía y demora en la atención (20, 31). A este repecto, dadas las restricciones al aborto legal en ALC, es probable que varios de los abortos inducidos atendidos en las instituciones de salud hayan sido clasificados como “incompletos” y que, por lo tanto, haya error en las estimaciones de la prevalencia para abortos tanto inducidos como espontáneos.

Lo anterior cobra aún más relevancia si se tiene en cuenta que ALC no ha alcanzado las metas de salud materna proyectadas por las agendas mundiales de desarrollo a pesar de que el acceso a los servicios institucionales obstétricos y perinatales ha vertebrado las estrategias para reducir la morbimortalidad materna. Garantizar la calidad y la aceptabilidad de la atención puede acrecentar la efectividad de dicho enfoque al aparejar el aumento en el acceso con el incremento en la utilización de los servicios y disminuir así la primera demora en la atención.

Según sabemos, esta es la primera revisión sistemática en estimar la prevalencia de irrespeto y maltrato en la atención institucional del parto y del aborto en ALC. Sin embargo, hemos encontrado varios desafíos en esta tarea. A pesar de la relevancia del fenómeno, solo cinco países han recabado evidencia al respecto y todos ellos pertenecen a América Latina, con lo cual los estudios encontrados fueron escasos y los resultados circunscritos con criterio geográfico.

Además, la mayoría de los estudios no contaron con representatividad nacional, pues los hospitales, en su mayoría públicos, fueron seleccionados sobre todo por conveniencia. Por otra parte, pocos estudios reportaron el tiempo transcurrido entre el parto o aborto y el momento de la entrevista, lo que da lugar a dudas sobre la introducción de sesgo de memoria (47). Asimismo, la realización de las entrevistas en el mismo establecimiento de salud donde las mujeres pudieron haber sufrido falta de respeto y maltrato, puede aumentar el riesgo de sesgo de cortesía y subestimar la prevalencia (47). Por otra parte, la diversidad que se presenta entre y dentro de los países con respecto a las construcciones culturales que determinan las percepciones de insatisfacción y maltrato en la atención, pueden incrementar la variabilidad de los resultados (43).

Por último, la carencia de datos no permitió realizar análisis sociodemográficos. Por lo tanto, no se exploró, por ejemplo, la ocurrencia del fenómeno entre mujeres en situaciones de vulnerabilidad -indígenas, afrodescendientes, migrantes, con discapacidad y adolescentes- reconociendo que estas poblaciones están expuestas a mayor riesgo de inequidad en la atención de la salud materna y que dicha inequidad se relaciona no solo con menor disponibilidad y acceso a los servicios, sino también con una calidad sistemáticamente desigual de estos.

## CONCLUSIONES

Los datos extraídos en esta revisión indican que el irrespeto y el maltrato durante la atención institucional del parto y del aborto son prevalentes en la Región. Sin embargo, dada la elevada heterogeneidad documentada en los estudios incluidos, no fue posible obtener una estimación de alta calidad sobre la magnitud regional del fenómeno. Los datos reunidos y analizados en este trabajo pueden incentivar la producción de investigaciones regionales con enfoque de salud pública que produzcan medidas representativas, rigurosas y comparables sobre este fenómeno. Esto es en especial relevante para la implementación de servicios de salud perinatal en general, y del parto y el aborto en particular, que sean aceptables y centrados en las mujeres.

Alcanzar esta meta requiere incorporar estrategias de calidad que apunten no solo a la implementación de servicios oportunos y seguros y a la mejora en las condiciones de los establecimientos y los sistemas de salud, sino que, en consonancia con las recomendaciones basadas en la evidencia, promuevan la participación de las mujeres en el diseño de los servicios, respondan a sus aspiraciones, necesidades y percepciones, cuenten con enfoques diferenciales y garanticen sus derechos a una atención respetuosa y libre de discriminación. Lo anterior es imprescindible para reducir la morbimortalidad materna e infantil, posibilitar que las mujeres, los niños y las niñas prosperen y alcancen su máximo potencial y contribuir, por ende, al desarrollo regional.

## Contribución de los autores.

AG y JPS concibieron el estudio; MP, CH, SM y VP seleccionaron estudios; MP extrajo los datos; CH y MP escribieron el manuscrito original; AG, LR, RVS, LAM, ARC, MPP, and RGPL revisaron el manuscrito. Todos los autores revisaron y aprobaron la versión final.

## Agradecimientos.

A Meghan Bohren, quien colaboró en el proceso de elaboración de las estrategias de búsqueda, y a Eleonora D’Orsi y la Fundación Perseu Abramo (en la persona de Matheus Toledo) por su valiosa colaboración.

## Declaración.

Las opiniones expresadas en este manuscrito son responsabilidad del autor y no reflejan necesariamente los criterios ni la política de la *RPSP/PAJPH* y/o de la OPS.
